# Disability Prevention Program Improves Life-Space and Falls Efficacy: A Randomized Controlled Trial

**DOI:** 10.1093/geroni/igaa057.848

**Published:** 2020-12-16

**Authors:** Minhui Liu, Qian-Li Xue, Laura Gitlin, Jennifer Wolff, Jack Guralnik, Bruce Leff, Sarah Szanton

**Affiliations:** 1 Central South University, Baltimore, Maryland, United States; 2 Johns Hopkins School of Medicine, Baltimore, Maryland, United States; 3 Drexel University, Philadelphia, Pennsylvania, United States; 4 Johns Hopkins Bloomberg School of Public Health, Baltimore, Maryland, United States; 5 University of Maryland School of Medicine, Baltimore, Maryland, United States; 6 Johns Hopkins University School of Medicine, Baltimore, Maryland, United States; 7 Johns Hopkins University, Baltimore, Maryland, United States

## Abstract

Life-space and falls efficacy are essential to physical independence and consistently predict disabilities. However, it remains unknown whether the CAPABLE, a disability prevention program, improves life-space and falls efficacy in older adults. We evaluated the effects of a home-based disability prevention program on life-space (N=194) and falls efficacy (N=233) among low-income older adults with restricted daily activities. The CAPABLE intervention consists of up to 6 one-hour home visits with an Occupational Therapist (OT), up to 4 one-hour home visits with a Register Nurse (RN), and up to $1300 worth of home repairs, modifications, and assistive devices with a handyman, during four months. Life-space and falls efficacy were measured by the Telephone Assessment of Mobility for homebound older adults and the Tinetti Falls Efficacy Scale at baseline and five months, respectively. Multinomial logistic regression and generalized linear models were used for data analyses. Participants in both samples were, on average, 75 years, predominantly black (86%) and female (85%-86%). Compared to participants in the control group, participants receiving the intervention were more likely to have improved (Odds Ratio [OR]: 2.10, 95% CI: 1.06-4.20) or unchanged overall life-space (OR: 2.62, 95% CI: 1.10-6.27) vs. decreased overall life-space. Participants who received the intervention also had significantly higher falls efficacy in performing daily activities (exponentiated coefficient: 1.11, 95% CI: 1.04-1.19). Life-space and falls efficacy can be significantly improved by CAPABLE program. These findings provide more evidence for the reasons for the increased physical independence.

